# Perceptual errors in Iranian society: A qualitative study 

**Published:** 2019-08

**Authors:** Ezat Samadipour, Hesam Seyedin, Hamid Ravaghi

**Affiliations:** ^*a*^Sabzevar Universuty of Medical Sciences, Sabzevar, Iran.; ^*b*^Iran university of Medical Sciences, Tehran, Iran.

## Abstract

**Background:**

General risk perception is defined as the collective wisdom of comprehension of risk, exposure and hazard in a society which decisions made during accidents and disasters are driven by the risk perceived by the influenced society. Always, the path of reaches to general perception is not smooth, one of the reasons of low impact risk reduction programs are perceptual errors that threat personal safety.

**Methods:**

This qualitative study was done considering the grounded theory. Thirty individuals participated in this study who were divided into two groups of expertise and ordinary people with maximum diversity. The data were collected through semi-structured interviews.

**Results:**

The finding of the study were extracted from 1357 primary codes. The risk perception structure in Iranian society was threatened with five diminishing factors called perceptual errors, negative experiences, negative beliefs, disapprovals and institutional factors. A perceptual error was due to an error in the estimation power of risk and error in its ability. The errors are less or excessive than the realistic estimated of the actual hazard. The individual's ability referred to coping power, therefore mistakenly estimates can cause personal injury. Some people consider denial or low-risk approach to their power to be sufficiently coped or dispersed. Negative experience occurs due to normalization of risk, and failure to predict incidence.

Risk perception is deliberately and voluntarily done. Non-acceptance is due to disclaimer of self-disclosure, conflict of interest. Institutional agents mean institutions that consist of governmental and non-governmental organizations that manage risk reduction. Some of institution agent are lack of commitment, trust, practical obligation and Instrumental use of people.

**Conclusions:**

The finding of this study can be identify perception errors that may play important role in actual understanding and create reasonable concern to providing precaution and safety. Actual risk perception formation need to educational, cultural and institutional strategies to promote risk perception. In order to paving up the safety upgrade path, policy makers, cultural, political and social managers can monitor and control this perception errors.

**Keywords:**

Risk perception, Risk reduction, Safety upgrade, Perception error, Iranian society

